# ACE-inhibition, but not weight reduction restores cardiomyocyte response to β-adrenergic stimulation in the metabolic syndrome

**DOI:** 10.1186/1471-2261-13-51

**Published:** 2013-07-12

**Authors:** Ines Nevelsteen, Virginie Bito, Gerry Van der Mieren, Annelies Vanderper, An Van den Bergh, Karin R Sipido, Kanigula Mubagwa, Paul Herijgers

**Affiliations:** 1 Department of Cardiovascular Sciences, Research Unit of Experimental Cardiac Surgery, KU Leuven, Herestraat 49, Leuven B-3000, Belgium; 2 Research Unit of Experimental Cardiology, KU Leuven, Leuven, Belgium

**Keywords:** Metabolic syndrome, Cardiomyocyte contractility, β-adrenergic stimulation, Hypocaloric diet, ACE-inhibition

## Abstract

**Background:**

Diabetic cardiomyopathy is characterized by systolic and early diastolic ventricular dysfunction. In the metabolic syndrome (MS), ventricular stiffness is additionally increased in a later stage. It is unknown whether this is related to intrinsic cardiomyocyte dysfunction, extrinsic factors influencing cardiomyocyte contractility and/or cardiac function, or a combination of both. A first aim was to study cardiomyocyte contractility and Ca^2+^ handling in vitro in a mouse model of MS. A second aim was to investigate whether in vivo hypocaloric diet or ACE-inhibition (ACE-I) improved cardiomyocyte contractility in vitro, contractile reserve and Ca^2+^ handling.

**Methods:**

This study was performed in LDL-receptor (LDLR−/−) and leptin-deficient (ob/ob), double knock-out mice (DKO), featuring obesity, type II diabetes, atherogenic dyslipidemia and hypertension. Single knock-out LDLR−/−, ob/ob and wild type mice were used as controls. Cellular contractility, Ca^2+^ handling and their response to in vivo treatment with diet or ACE-I were studied in isolated cardiomyocytes at baseline, during β-adrenergic stimulation or increased extracellular Ca^2+^, using field stimulation and patch-clamp.

**Results:**

In untreated conditions, prolongation of contraction-relaxation cycle and altered Ca^2+^ handling are observed in MS. Response to increased extracellular Ca^2+^ and β-adrenergic stimulation is impaired and could not be rescued by weight loss. ACE-I restored impaired response to β-adrenergic stimulation in MS, but not the decreased response to increased extracellular Ca^2+^.

**Conclusions:**

Cardiomyocyte contractility and β-adrenergic response are impaired in MS, due to alterations in cellular Ca^2+^ handling. ACE-I, but not weight loss, is able to restore cardiomyocyte response to β-adrenergic stimulation in MS.

## Background

The metabolic syndrome (MS) is characterized by the co-occurrence of at least three of the following features: abdominal obesity, hypertension, dyslipidemia, insulin resistance ± glucose intolerance, pro-inflammatory and pro-thrombotic status. The prevalence of MS and diabetes mellitus type II (DMII) is increasing. Affected patients are at higher risk of developing cardiovascular complications, caused by diabetic, combined with ischemic, cardiomyopathy and angiopathy. The existence of a primary diabetic cardiomyopathy has been shown in diabetic animal models [1]. The co-occurrence of hypertension and dyslipidemia in DMII aggravates cardiovascular dysfunction in vivo [2].

MS-associated cardiomyopathy can be distinguished in vivo from diabetic cardiomyopathy by impaired late diastolic function, lower end-diastolic volume, decreased cardiac output and concentric hypertrophy [2].

Cardiac function is determined by intrinsic cardiomyocyte function, but also influenced by myoarchitecture, composition and structure of the extracellular matrix, by preload and afterload. It is unclear whether the in vivo features of heart failure in MS are caused by intrinsic cardiomyocyte dysfunction and/or extrinsic factors influencing cardiomyocyte or cardiac contractility. No studies are available on isolated cardiomyocyte contractility in MS-mice.

Calcium plays a key role in excitation-contraction coupling in cardiomyocyte contractility. Myocyte contraction is determined by entry of Ca^2+^ via L-type calcium channels that triggers release of Ca^2+^ from the sarcoplasmatic reticulum (SR) via ryanodine receptors (RyR) and activation of myofilaments. Relaxation occurs with sequestration of Ca^2+^ from the cytosol to the SR by SR Ca^2+^ ATP-ase (SERCA2a) and extrusion of Ca^2+^ out of the cell by the sodium-calcium exchanger (NCX). Alterations in Ca^2+^ cycling in DMII and failing hearts have been extensively studied [3-5]. We have shown previously in ventricular homogenates of MS-mice that phosphorylation of phospholamban (PLB) is decreased and associated with an increased SERCA2a-expression [2]. Although these data provide insights into contributing mechanisms to contractile dysfunction in vivo, changes in intrinsic properties of cardiomyocytes have not been studied in MS-mice.

Diet and ACE-I are the golden standard in the treatment of DMII and MS. Their beneficial effects on cardiovascular complications have been established in clinical and experimental settings. Diet has proven to reduce cardiac hypertrophy, to ameliorate diastolic function, insulin resistance and blood pressure in MS [6-8]. ACE-I improves endothelial function, reverses cardiovascular remodeling effects and has an anti-atherosclerotic effect [9-11]. It remains unclear to what extent these observations can be explained by intrinsic cardiomyocyte dysfunction and/or extrinsic factors influencing cardiomyocyte or cardiac contractility.

The first aim of our study was to phenotype cardiomyocyte contractility and Ca^2+^ handling in DKO to gain further insight in the contributing mechanisms to cardiac failure in MS. A second goal was to study the effect of in vivo treatment with diet or ACE-I on cardiomyocyte contractility and Ca^2+^ handling.

## Methods

### Animals

Experiments were conducted in homozygous LDL-receptor (LDLR−/−) and leptin-deficient (ob/ob), double knock-out mice (DKO) of either gender at 12, 18 and 24 weeks with C57BL/6J (WT), LDLR−/− and ob/ob as reference data. Mice were backcrossed for at least 10 generations into C57BL/6J, exhibiting 98.4% C57BL/6J background. Heterozygous ob/+ and C57BL/6J were purchased from Jackson Laboratory (Bar Harbor, ME, USA). Homozygous ob/ob and DKO mice were generated as described previously [12]. The investigation conforms to the *Guide for the Care and Use of Laboratory Animals* published by the US National Institutes of Health (NIH Publication No.85-23, revised 1996) and approved by the institutional review board.

### Metabolic analysis

Blood was taken by tail bleeding into EDTA-tubes, after 24 hours fasting.

Serum glucose was measured with a glucometer (Menarini Diagnostics). Plasma was acquired by centrifugation. Triglycerides (TGL), total cholesterol (Chol), high-density lipoprotein (HDL) and low-density lipoprotein levels (LDL) were determined with a diagnostic reagent kit (Roche).

### Food restriction

Mice were fed standard chow containing 9% fat (Ssniff, Germany). Food intake of free-fed DKO and LDLR−/− mice was respectively 5.7 and 2.5 g/day [13]. Food intake of diet-restricted DKO and ob/ob was reduced to the daily food intake of LDLR−/− (2.5 g/day) with free access to water, between 12 and 24 weeks of age.

### ACE-inhibition

Captopril (10 mg/kg) [14] was daily injected intraperitoneally (from 12 till 24 weeks of age) in ob/ob and DKO.

### Cell isolation

Ventricular myocytes from 24 weeks-old mice were isolated as described previously [15]. Briefly, mice were heparinized (5000 IU/ml) and euthanized with pentobarbital (50 mg/ml, ip). The heart was excised and weighted. Single left ventricular myocytes were obtained by enzymatic dissociation through retrograde perfusion of the aorta, using collagenase A (0.4-0.5 mg/ml, Roche Diagnostics, Germany) and protease (type XIV 0.08 mg/ml, Sigma, USA). Cells were used within 5 hours after isolation.

### Measurement of cellular contraction, [Ca^2+^]_i_ transients (CaT) and SR Ca^2+^ content

Unloaded cell shortening was measured by video-edge detection (Crescent, Salt Lake City, USA) during field stimulation (Myopacer Cell Stimulator, IonOptix, Milton, USA). Cells were stimulated at 1, 2 and 4 Hz to determine a shortening-frequency relationship. Cell contraction is expressed as fractional cell shortening (FCS) i.e. cell length change normalized to resting cell length. Kinetics of contraction were evaluated as time-to-peak contraction (TTP) and relaxation as time to 50% relaxation (RT50), starting from peak contraction.

CaT were measured in cardiomyocytes, preloaded with Fluo-3 AM (10 μM for 20 minutes), using perforated patch-clamp and data were calibrated as previously described [16]. SR Ca^2+^ load was estimated as the peak CaT obtained during 7s fast application of 10 mM caffeine. NCX function was evaluated as the decline of the caffeine evoked CaT. In a subset of cells, ruptured patch-clamp was used to asses L-type Ca^2+^ current (I_CaL_). The voltage-clamp protocol consisted of a pre-step from −70 to −50 mV to inactivate I_NaL_, followed by depolarizing voltage steps from −40 up to +60 mV; duration of the step was 200 ms. Pipette solution was Cs-aspartate based with 10 mM NaCl.

The study on Ca^2+^ handling was performed in untreated and food-restricted DKO using WT as control.

### Response to Ca^2+^ challenge and β-adrenergic stimulation

Contractile reserve was studied at 1 Hz by using isoproterenol (50 nM) (non-selective β-adrenoreceptor agonist) or by increasing extracellular Ca^2+^ (from 1 to 1.8 mM). FCS was measured at steady-state and expressed as percent increase in FCS.

All experiments were performed at 35°C.

### Data management and statistical analysis

Analysis of data was performed using Clampfit 8.2 (Axon Instruments, Scotland), Origin (Originlab, USA) and Ionwizard (IonOptix, USA). Data are expressed as mean ± standard error of the mean (SEM).‘N’ denotes number of animals and ‘n’ denotes number of cells. Differences between groups were analyzed for statistical significance by ANOVA with a Fisher post-hoc test, using Statistica 7.1 (StatSoft, USA). p < 0.05 was considered significant.

## Results

We did not see any difference between WT and LDLR−/−. Therefore, results obtained from LDLR−/− will not be reported. Additionally, ACE-I did not alter in vivo cardiac contractility in WT [17]. Therefore, cardiomyocyte contractility of WT after ACE-I has not been studied.

### Phenotypic and metabolic evolution (Table 1)

#### *Phenotypic and metabolic features of MS in untreated DKO*

At 24 weeks, DKO and ob/ob body weight (BW) was significantly higher, compared to WT. Similarly, heart weight (HW) was higher in DKO and ob/ob, resulting in a significantly lower heart weight over body weight ratio (HW/BW ratio), compared to WT. DKO and ob/ob showed a significant hyperglycemia. Additionally, DKO showed a marked atherogenic dyslipidemia (high levels of Chol, TGL and LDL), compared to WT and ob/ob.

**Table 1:** Metabolic and phenotypic evolution

	**WT**	**Ob**/**ob**			**DKO**		
**At 12 w**		**Untreated**	**Food restriction**	**ACE**-**I**	**Untreated**	**Food restriction**	**ACE**-**I**
Body Weight(g)	21.1±1.0	49.5±0.6*	49.8±0.9*	49.0±0.3*	48.8±0.7*	51.2±0.6*	45.2±1.6*
Glycemia(mg/dL)	56±2.0	159.3±18.5^*^	166±13.7*	155±14.5*	155±10.2*	156±8.8*	160±12.9*
Total Chol(mg/dL)	104±25	129±6^£^	131±6	116±6	977±45*^+^	958±77*	735±90*^£^
Triglycerides(mg/dL)	115±15	53±2*^£^	53±2*	69±3*	585±66^*+^	618±75*	455±69*
HDL(mg/dL)	68±11	106±5*^£^	107±5*	90±4*	232±5*^+^	112±10*^£^	137±11*^£^
LDL(mg/dL)	16±11	13±2	14±2	12±3	/	/	308±228^*^
	**WT**	**Ob**/**ob**			**DKO**		
**At 18 w**		**Untreated**	**Food restriction**	**ACE**-**I**	**Untreated**	**Food restriction**	**ACE**-**I**
Body Weight(g)	22.1±1.3	56.6±0.9^*£^	37.1±1.1*^+^	54.0±0.4*	49.8±3.4*^+^	37.3±0.7*^£^	53.9±1.8*
Glycemia(mg/dL)	72±4.5	134±18.9	176±19.9*	119±9.1	142±13.4	158±19.8*	191±24.3*
Total Chol(mg/dL)	80±7	154±13*	77±6^+^	140±10*	579±145*^+^	794±59*	527±63*
Triglycerides(mg/dL)	75±6	81±2	74±5	70±2	334±80*^+^	331±38*	171±19^£^
HDL(mg/dL)	62±6	123±9^*£^	67±7^+^	112±7*	100±6*^+^	201±18*^£^	159±11*^£^
LDL(mg/dL)	4±2	15±4*	/	16±3*	277±100*^+^	519±35*^£^	334±50*
	**WT**	**Ob**/**ob**			**DKO**		
**At 24 w**		**Untreated**	**Food restriction**	**ACE**-**I**	**Untreated**	**Food restriction**	**ACE**-**I**
Body Weight(g)	27.1±0.9	64.5±0.9^*^	34.0±2.3*^+^	65.8±1.3*	61.2±1.7*	35.9±1.3*^£^	61.8±1.0*
Heart Weight(mg)	164.4±9.6	196.1±9.3*	136.4±2.8*^+^	178.7±4.0	184.2±10.0	132.7±3.0*^£^	161.2±4.0^£^
HW/BW ratio(mg/g)	6.2±0.5	3.1±0.1^*^	4.3±0.3*^+^	2.7±0.1^*^	3.1±0.1^*^	3.8±0.2^*^	2.6±0.1^*^
Glycemia(mg/dL)	71±4,5	166±11.8*	142±12.1*	84±5.0^+^	141±28.6*	153±18.2*	124±8.7*
Total Chol(mg/dL)	93±17	139±10	74±8	145±10	576±78*^+^	816±74*^£^	634±55*
Triglycerides(mg/dL)	68±9	68±3	69±5	76±4	210±30*^+^	301±60*	202±21*
HDL(mg/dL)	73±9	97±6	66±8	100±2	183±15*^+^	232±21*^£^	192±4*
LDL(mg/dL)	8±7	29±5	/	34±8	351±66*^+^	432±24*	402±49*

This table shows the metabolic and phenotypic evolution from 12 till 24 weeks of age of WT (N =13), untreated ob/ob (N =10), untreated DKO (N =13), food-restricted ob/ob (N =32), food-restricted DKO (N =19), ob/ob treated with ACE-I (N =14) and DKO treated with ACE-I (N =16).

* denotes p < 0.05 vs. WT, + p < 0.05 vs. untreated ob/ob and £ p < 0.05 vs. untreated DKO; *HW* heart weight, *BW* body weight, *Total Chol* total cholesterol, *HDL* high-density lipoprotein, *LDL* low-density lipoprotein and / = No data available.

#### *Significant weight loss with hypocaloric diet*

Diet resulted in significant weight loss in DKO and ob/ob, associated with significantly lower HW. HW/BW ratio was significantly increased in ob/ob after diet, compared to untreated ob/ob. Less marked effect of diet on HW/BW ratio was obtained in DKO. Hyperglycemia was reduced in ob/ob, whereas hyperglycemia and atherogenic dyslipidemia were still present in DKO after diet. The same was observed at 12 and 18 weeks.

#### *Significantly lower heart weight in DKO after in vivo treatment with ACE-I*

ACE-I resulted in a significantly lower HW in DKO, but unchanged BW, leading to a small decrease in HW/BW ratio. Neither HW, nor BW were influenced by ACE-I in ob/ob. Fasting glycemia was no longer increased in ob/ob whereas hyperglycemia and atherogenic dyslipidemia remained present in DKO after ACE-I.

### Cardiomyocyte contractility in vitro

#### *Prolongation of excitation-contraction cycle in untreated DKO*

**Figure 1:**
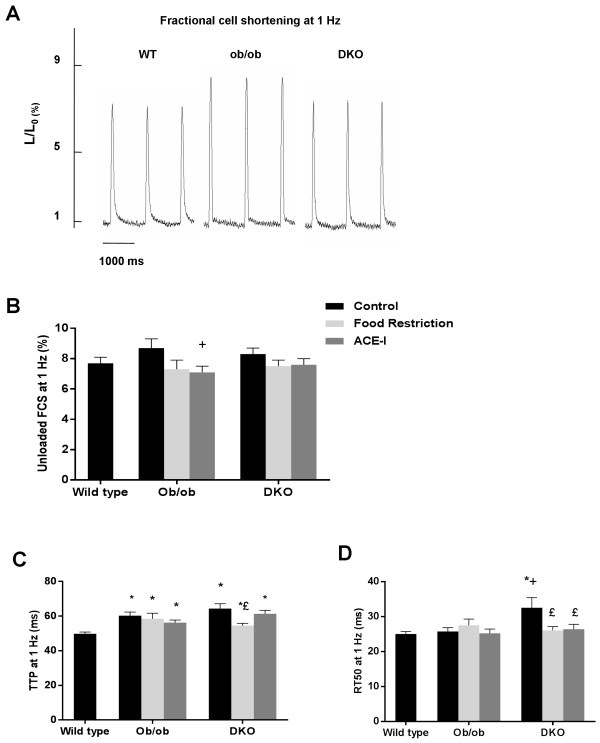
**Cell contraction at baseline in WT, ob/ob and DKO at 24 weeks.** Representative example of unloaded cell shortening at 1 Hz in WT, ob/ob and DKO **(A)**, unloaded cell shortening **(B)**, TTP **(C)** and RT50 **(D)** at 1 Hz stimulation frequency in WT (n = 44), ob/ob (n = 24) and DKO (n = 25). * denotes p < 0.05 vs. WT, + p < 0.05 vs. untreated ob/ob and £ p< 0.05 vs. untreated DKO.

**Figure 2:**
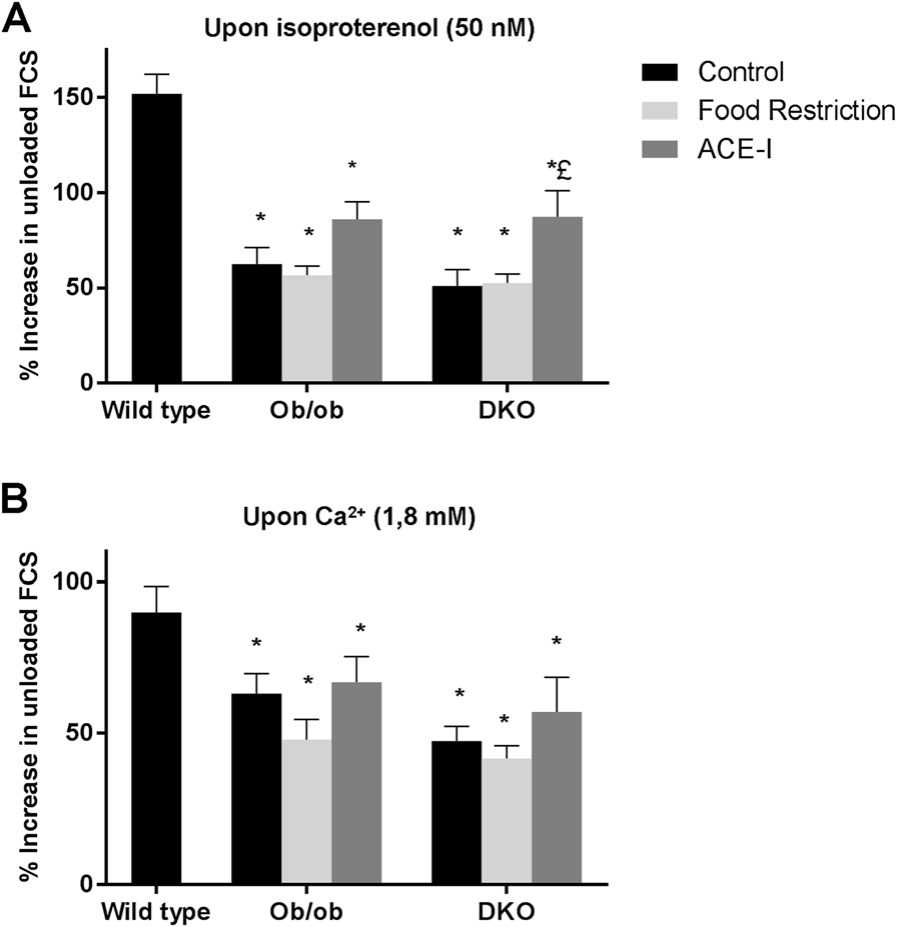
**Myocyte contraction under stress in the different genotypes and effect of treatment at 24 weeks.** Effect of 50 nM isoproterenol on cell shortening amplitude **(A)** and effect of increased extracellular Ca^2+^ (1.8 mM) on cell shortening amplitude **(B)** in the different groups. * denotes p < 0.05 vs. WT, + p < 0.05 vs. untreated ob/ob and £ p < 0.05 vs. untreated DKO.

A similar, negative shortening-frequency relationship was observed in DKO, WT and ob/ob (data not shown). Cell width was significantly larger in DKO (13.4±0.3 μm) and ob/ob (13.5±0.3 μm) compared to WT (9.5±0.2 μm), with similar resting cell length in all groups (data not shown). Figure 1A is a representative example of unloaded cardiomyocyte shortening at 1 Hz in WT, ob/ob and DKO with similar FCS in all genotypes (Figure 1B). TTP was significantly longer in DKO and ob/ob than in WT (Figure 1C). RT50 was significantly longer in DKO, than in WT and ob/ob (Figure 1D). Regarding contractile reserve, cell shortening increased in all genotypes upon isoproterenol application or increased extracellular Ca^2+^. However, the relative increase was in both situations smaller in ob/ob and further reduced in DKO, compared to WT (Figure 2).

#### *Normalization of contraction cycle in DKO after diet*

Diet improved contraction and relaxation in DKO only; TTP and RT50 were significantly shorter after diet in DKO, compared to untreated DKO (Figure 1C and D). However, the response to isoproterenol or increased Ca^2+^ remained impaired in DKO, as well as in ob/ob (Figure 2).

#### *Partial rescue of contractile reserve after in vivo treatment with ACE-I*

DKO developed a significantly faster relaxation after ACE-I, but TTP was unaffected (Figure 1C and D). Unlike diet, ACE-I leads to a significant increase in β-adrenergic response in DKO, compared to untreated DKO. Response to extracellular Ca^2+^ was not improved. In ob/ob, there was a clear tendency towards improvement of the response to isoproterenol or increased Ca^2+^, without reaching significance (Figure 2).

### Calcium handling in isolated cardiomyocytes

#### *Impaired Ca^2+^ handling in untreated DKO*

**Figure 3:**
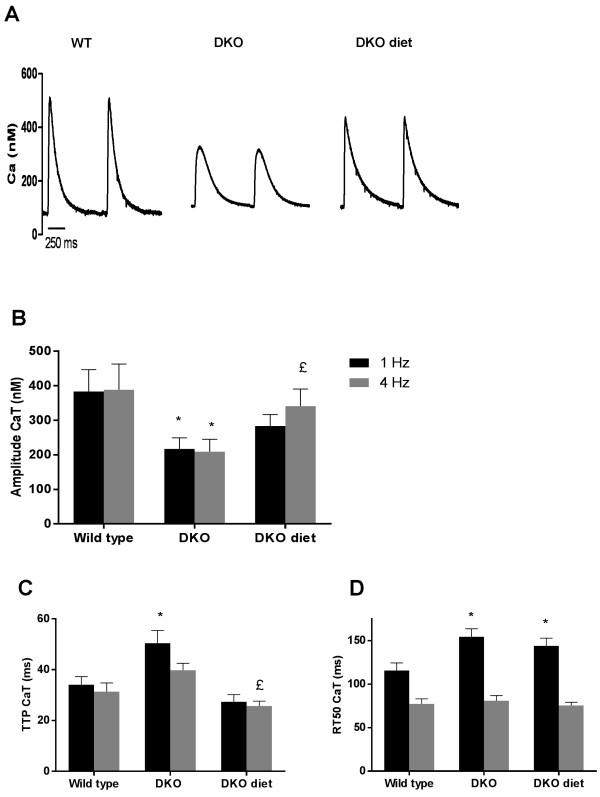
**CaT properties in WT, DKO and DKO undergoing hypocaloric diet. (A)** Representative example of CaT recording during short depolarizing pulses at 1 Hz in WT, DKO and DKO after diet. CaT amplitude **(B)** time to peak **(C)** and RT50 **(D)** in WT ( n = 14), DKO (n = 21) and DKO diet (n = 18). * denotes p < 0.05 vs. WT and £ p < 0.05 vs. untreated DKO.

**Figure 4:**
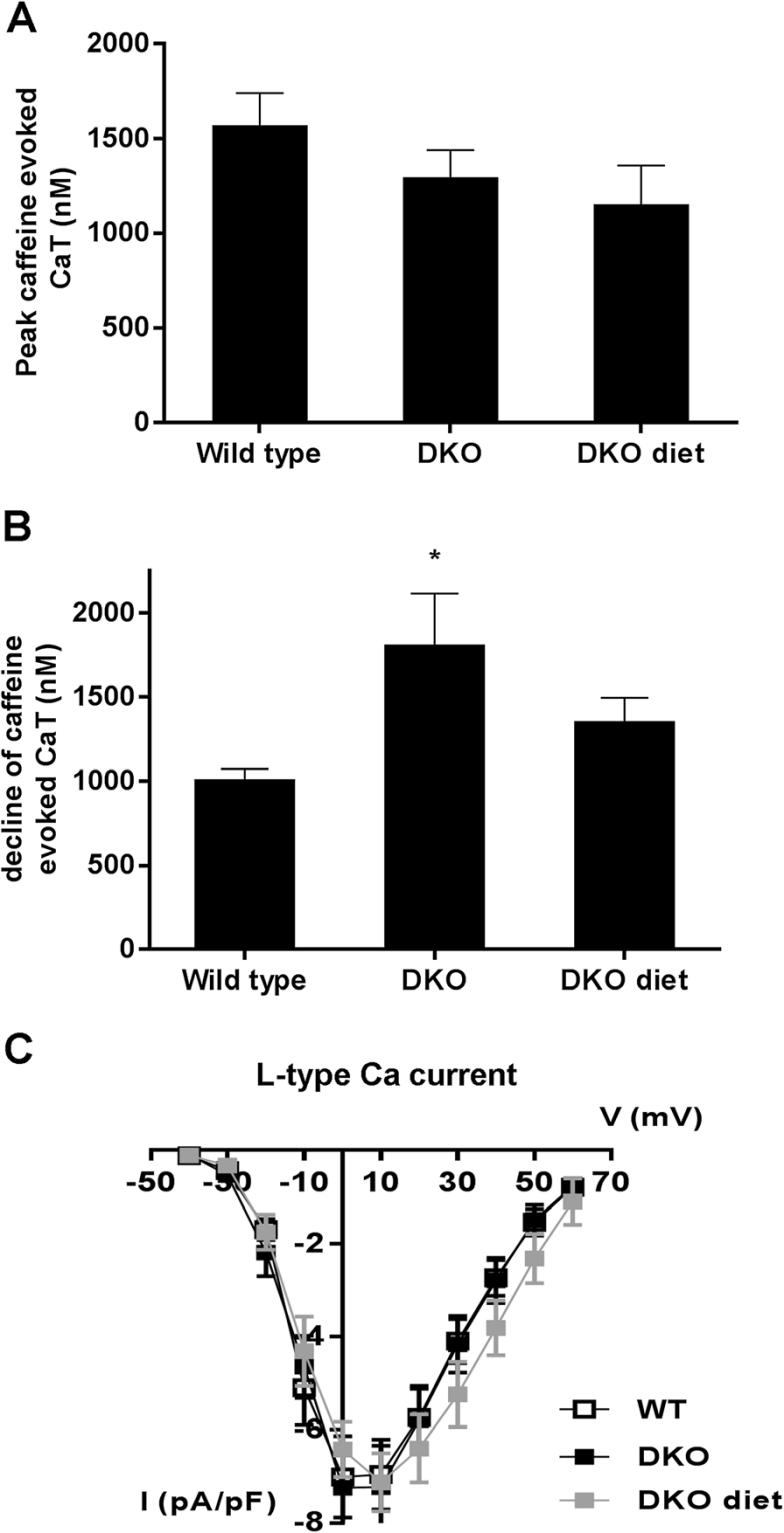
**SR Ca**^**2+ **^**content, NCX function and I**_**CaL **_**in WT, DKO and DKO undergoing hypocaloric diet. (A)** Averaged data on SR Ca^2+^ content, measured as the peak caffeine-evoked CaT in WT (n=12), DKO (n =12) and DKO diet (n=12). **(B)** Pooled data on NCX function, estimated as the decline of the caffeine-induced CaT in WT (n=12), DKO (n=12) and DKO diet (n=12). **(C)** L-type Ca^2+^ current density (ICaL) expressed as a function of voltage in WT (n=11), DKO (n=8) and DKO diet (n=10). * denotes p<0.05 vs WT and £ p<0.05 vs. untreated DKO.

Figure 3A shows a representative example of CaT obtained during short depolarizing pulses at 1 Hz in WT and DKO. Diastolic Ca^2+^ at 0.5, 1, 2 and 4 Hz was not different between WT and DKO (data not shown). At 1 and 4 Hz, CaT amplitude in DKO was significantly smaller (Figure 3B), associated with slower kinetics (longer TTP and slower relaxation) (Figure 3C and D), compared to WT. SR Ca^2+^ content, evaluated as the peak CaT evoked during caffeine application was not different between DKO and WT (Figure 4A). Decline of the caffeine evoked CaT was prolonged in DKO compared to WT (Figure 4B). I_CaL_ density and voltage-dependence were similar for WT and DKO (Figure 4C).

#### *Improved Ca^2+^ handling in DKO after diet*

Diet resulted in a significant increase in CaT amplitude in DKO with faster kinetics, in particular TTP (Figure 3). SR Ca^2+^ content remained comparable between the groups after diet (Figure 4A). Ca^2+^ decline during caffeine application was improved with diet (Figure 4B). I_CaL_ remained unchanged after diet (Figure 4C).

## Discussion

In this study, we determined cardiomyocyte contractility and Ca^2+^ handling in isolated cardiomyocytes in a mouse model of MS. The first new finding of our study is functional characterization of single cardiomyocytes in a mouse model of MS. At the cellular level, MS is characterized by slower kinetics of contraction and relaxation. DKO mice show a significantly longer TTP and RT50, indicating prolongation of contraction-relaxation cycle. The second finding is that contractile reserve is impaired in isolated cardiomyocytes from MS-mice. ACE-I, but not weight reduction, can restore the response to β-adrenergic stimulation. However, the attenuated response to extracellular Ca^2+^ is not improved by the aforementioned treatments.

Both findings are a hallmark of failing myocardium. Furthermore, these cellular defects are in parallel with previous in vivo studies in our laboratory and with clinical studies [1,2,6-8].

### Diastolic dysfunction

In vivo, diastolic dysfunction in MS is characterized by an early and late component. The late component is due to enhanced myocardial fibrosis, resulting in decreased end-diastolic volume [2]. The early component is characterized by impaired active relaxation. Early relaxation occurs when Ca^2+^ is removed from the cytoplasm into the SR via SERCA2a and out of the cell by the NCX, and reflects dissociation of Ca^2+^ from contractile myofilaments. SERCA2a is a membrane protein that catalyzes ATP-dependent transport of Ca^2+^ from the cytosol into the SR and regulated by PLB. In 24 weeks-old DKO, preserved PLB/SERCA2a ratio is associated with decreased phosphorylation of PLB, resulting in decreased activity of SERCA2a [2]. NCX is considered as the second most important mechanism for removing Ca^2+^ from the cytosol. Hattori et al. showed impaired NCX function in type I DM (DMI), due to decreased protein expression, playing an important role in altered Ca^2+^ handling [18]. In DKO, we report a significantly longer RT50 for cardiomyocyte contraction and CaT, mainly related to SERCA2a and PLB. Ca^2+^ decline after administration of caffeine is significantly prolonged in DKO, indicating altered NCX function. Although, we did not look at changes in NCX expression, it is likely that both mechanisms (decreased phosphorylation of PLB and altered NCX function) contribute to impaired relaxation in MS. Finally, unaltered levels of resting calcium indicate that the “pump-leak balance” is unchanged, as previously described in DMI [4].

After in vivo treatment with diet and ACE-I, RT50 is comparable with WT. Diet also improved Ca^2+^ handling in DKO. These findings are consistent with previous in vivo studies, showing diet to improve cardiac function and insulin sensitivity in MS-mice [5-7]. The beneficial effect of ACE-I is largely due to improved endothelial function, its anti-atherosclerotic and anti-remodeling effects [9-11,19]. All together, our data provide evidence for contribution of intrinsic cellular mechanisms, in recovering diastolic function after diet and ACE-I, associated with extrinsic mechanisms (eg. reduced ventricular-vascular stiffening, reduced subendothelial lipid deposition and increased endothelium-dependent relaxation) [6-11,19].

### Systolic dysfunction

In vivo, MS is characterized by systolic dysfunction, reflected by reduced cardiac output [2]. Cellular contraction starts when Ca^2+^ enters the cell via L-type Ca^2+^ channels and thereby activating RyR that will lead to further Ca^2+^ release from the SR. Ca^2+^ will in turn activate myofilaments, inducing contraction. This process is known as calcium-induced-calcium-release, playing a key role in cellular contraction.

In DMI, L-type Ca^2+^ channel activity is unchanged, but RyR protein levels are decreased [4]. DKO have a significantly smaller CaT amplitude and a significantly larger TTP. These data, taken together with lower protein levels of RyR, suggest RyR to be likely responsible for the slow Ca^2+^ release from the SR, since no changes in L-type Ca^2+^ current were found. Our data in DKO show that FCS is preserved despite a decrease in CaT amplitude. This might be explained by an increase in myofilament Ca^2+^ sensitivity. However, we did not asses myofilament sensitivity in DKO and experiments on contractility and Ca^2+^ handling were conducted in different cell groups. It is also unclear how DM affects myofilament sensitivity. Whereas some studies suggested that DM is associated with decreased myofilament sensitivity, others indicate the opposite [20,21]. On the other hand, it is important to notice that slowed relaxation, as found in DKO, is also a feature of increased myofilament sensitivity. Diet and ACE-I did not influence TTP of cellular contraction. In contrast, diet did improve kinetics of CaT in DKO, in particular TTP, suggesting that diet might influence expression or activity of RyR or myofilament sensitivity.

### Decreased β-responsiveness

Depressed β-responsiveness is a hallmark of heart failure. The mechanisms underlying impaired β-responsiveness have been extensively studied. It remains unclear whether this attenuated response is attributable to decreased expression of β-receptors, to distal transduction pathways defects and/or to impaired Ca^2+^ handling. Previous studies showed a decreased β-receptor expression/activity; however, more recent studies demonstrate impaired β-inotropy, without changes in β-adrenoceptor expression/activity [22,23]. Minhas et al. showed impaired β-adrenergic response in ob/ob, due to altered PKA-activity and reversible by leptin repletion [24]. We confirmed impaired contractile reserve in ob/ob and found a more pronounced impairment in DKO. Although we did not study β-receptor expression/activity or distal signaling pathways, it is likely that altered PKA-activity might also play a role in DKO, due to the same lack of leptin in these mice. Another possible contributor is reduced insulin sensitivity. As shown, DKO are hyperglycemic, hyperinsulinemic and insulin resistant. Insulin might contribute to cardiomyocyte β-responsiveness because of cross-talk between β-adrenergic and insulin receptors, mediated via Akt-pathway [25]. Consistent with this cross-talk, overexpression of insulin growth factor improves β-response in DMI [26]. On the other hand, insulin treatment in animals with DMI did not alter PKA-mediated activity and phosphorylation [27]. Whether insulin resistance contributes to β-inotropic hyporesponsiveness remains controversial. Yonemochi et al. showed in cultured ventricular neonatal rat myocytes that captopril enhances β-adrenergic responsiveness by inducing β-adrenergic receptor upregulation, mediated via activation of bradykinin B2-receptors and PKC [28].

Our study gives more insight in the pathophysiological mechanisms contributing to heart failure in MS and provides potential therapeutic targets in prevention and treatment of cardiovascular complications in MS.

### Limitations of our study

A limitation of our study is leptin deficiency in our mice. In humans, obesity is associated with hyperleptinemia. Long-term hyperleptinemia results in leptin resistance. Leptin resistance and interrupted leptin signaling were reported in cardiomyocytes under hyperleptinemic conditions, making hyperleptinemia comparable to leptin deficiency regarding signaling pathways of leptin [29].

A possible contributor to our results after diet is the extreme weight loss that our mice experienced. It was remarkable that heart weight in DKO and ob/ob after diet was lower, compared to WT. Extreme weight loss eg. starvation, has detrimental effects on cardiac performance [30]. These adverse starvation-effects strongly emphasize the need for tightly controlled weight reduction.

In the present study, we did not study the effect of ACE-I or β-adrenergic stimulation on Ca^2+^ handling, neither did we investigate molecular changes in calcium handling or contractile proteins. These remain interesting issues for future research.

## Conclusion

In vivo cardiac dysfunction, as seen in MS and shown previously in our laboratory and in humans, can at least partially be explained by alterations of intrinsic properties of cardiomyocytes [1,2,8,9]. We have shown that cardiomyocyte contractility and β-adrenergic response are impaired in MS, due to alterations in Ca^2+^ handling. ACE-I, but not weight loss, can restore impaired cardiomyocyte response to β-adrenergic stimulation in MS-mice.

## Abbreviations

ACE-I: Angiotensin-converting-enzyme inhibition; CaT: Calcium transient; DKO: Double knock-out mouse; DMI: Diabetes Mellitus type I; DMII: Diabetes Mellitus type II; FCS: Fractional cell shortening; LDLR−/−: Low-density lipoprotein receptor knock-out mouse; NCX: Sodium-calcium exchanger; Ob/ob: Leptin-deficient mouse; PLB: Phospholamban; RT50: Time to 50% relaxation; RyR: Ryanodin receptor; SERCA2a: Sarcoplasmatic Ca^2+^ ATP-ase; SR: Sarcoplasmatic reticulum; TTP: Time-to-peak contraction; WT: Wild type mouse.

## Competing interests

The authors declare that they have no competing interests.

## Authors’ contributions

IN participated in the conception and design of the study, data acquisition, (statistical) analysis and interpretation of the results and drafting of the manuscript. IN obtained funding for this study (see Acknowledgements). VB participated in acquisition and analysis of data and was involved in drafting the manuscript. GVDM, AVDP, AVDB helped with acquisition and interpretation of data on phenotypic and metabolic characteristics, as well as the effect of treatment on these chraracteristics. GVDM, AVDP, AVDB critically revised the manuscript. KM, KRS and PH participated in the conception and design of the study. KM and KRS gave a critical revision of the manuscript for important intellectual content. PH participated in the conception and design of the study and in statistical analysis of the data. PH obtained funding for this project (see Acknowledgements) and was involved in drafting and finalizing the manuscript. All authors have read and approved the final manuscript.

## Pre-publication history

The pre-publication history for this paper can be accessed here:

http://www.biomedcentral.com/1471-2261/13/51/prepub
